# Atypical Avian Influenza (H5N1)

**DOI:** 10.3201/eid1007.040415

**Published:** 2004-07

**Authors:** Anucha Apisarnthanarak, Rungrueng Kitphati, Kanokporn Thongphubeth, Prisana Patoomanunt, Pimjai Anthanont, Wattana Auwanit, Pranee Thawatsupha, Malinee Chittaganpitch, Siriphan Saeng-Aroon, Sunthareeya Waicharoen, Piyaporn Apisarnthanarak, Gregory A. Storch, Linda M. Mundy, Victoria J. Fraser

**Affiliations:** * Thammasart University Hospital, Pratumthani, Thailand;; † National Institute of Health, Nonthaburi, Thailand;; ‡ Siriraj Hospital, Bangkok, Thailand;; § Washington University School of Medicine, St. Louis, Missouri, USA

**Keywords:** Avian influenza, influenza, H5N1, healthcare workers, unusual presentation, Thailand, dispatch

## Abstract

We report the first case of avian influenza in a patient with fever and diarrhea but no respiratory symptoms. Avian influenza should be included in the differential diagnosis for patients with predominantly gastrointestinal symptoms, particularly if they have a history of exposure to poultry.

Influenza A viruses are classified into subtypes (hemagglutinin and neuraminidase subtypes) based on antigenic differences in their surface glycoproteins (1). Of 15 identified hemagglutinin (H1–H15) and 9 neuraminidase subtypes (N1–N9), only 3 hemagglutinin subtypes (H1, H2, and H3) and 2 neuraminidase subtypes (N1 and N2) have established stable lineages in humans (1). Because the natural reservoir of known influenza A subtypes is found in birds and waterfowl (2), subtypes other than those typically found in humans have the potential to cross the species barrier and infect humans (3).

Avian influenza A virus H9N2 was isolated from two children in Hong Kong in 1999, and avian influenza H7N7 infected 89 persons during a simultaneous outbreak in poultry in the Netherlands in 2003 (4–7), although these infections resulted in only mild illnesses. The first outbreak of a highly pathogenic avian influenza (H5N1) in humans occurred in Hong Kong in 1997; 6 of 18 people with confirmed infection died (5,8). Despite attempts to prevent disease, two cases of influenza A H5N1 occurred in Hong Kong in February 2003 (1), followed by outbreaks in Vietnam and Thailand in January 2004 (9,10). Data are limited on the epidemiologic characteristics, signs and symptoms, and outcomes of avian influenza H5N1 exposure in healthcare workers. We report atypical avian influenza H5N1 and follow-up surveillance of 35 exposed healthcare workers; we also review relevant literature in this area.

## The Case

**Figure 1:**
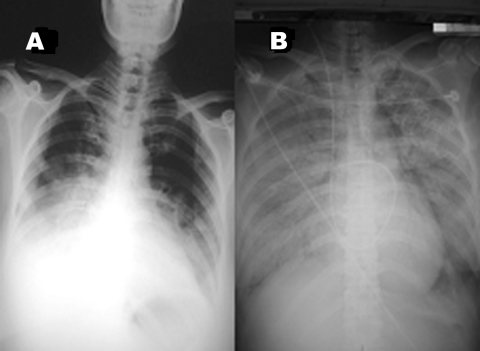
A) Chest radiograph on hospital day 5 at referring hospital shows patchy infiltration at bilateral lower lung fields. B) Chest radiograph upon admission to our hospital (24 hours later) shows rapidly progressive pneumonia in both lung fields, compatible with adult respiratory distress syndrome.

On March 9, 2004, a 39-year-old woman with no underlying disease was transferred to our hospital with rapidly progressive pneumonia. At the referring hospital, she reported fever for 1 week, diarrhea, nausea, and vomiting, with no early respiratory symptoms. Initial laboratory values at the referring hospital included the following: leukocyte count 3,300 cells/mm^3^, total lymphocyte count 640 cells/mm^3^, hemoglobin 13 g/dL, and platelet count 400,000 cells/mm^3^. Stool samples and cultures were negative for bacteria and parasites. Because the patient had no respiratory symptoms, no chest radiograph was performed on initial admission. Norfloxacin was prescribed. On hospital day 5, cough and shortness of breath developed, and chest radiograph was performed (Figure 1A). Norfloxacin was changed to ceftazidime and amikacin, and the patient was transferred.

Upon arrival at our hospital, her temperature was 39.4°C, respiratory rate 44/min, blood pressure 110/80 mm Hg, and heart rate 140 beats/minute. She was intubated, and the examination showed bilateral crackles. Laboratory data included leukocyte count 2,200 cells/mm^3^ with total lymphocyte counts of 440 cells/mm^3^, hemoglobin 11.1 g/dL, platelet count 330,000 cells/mm^3^, aspartate aminotransferase 474 U/L, alanine aminotransferase 106 U/L, alkaline phosphatase 546 U/L, blood urea nitrogen 11 mg/dL, creatinine 1.6 mg/dL, partial thromboplastin time 35.4 s, prothrombin time 11.9 s, and lactase dehydrogenase 1,832 mg/dL. Imipenem, azithromycin, and doxycycline were administered as adult respiratory distress syndrome progressively developed. Infectious disease consultation was requested the next day, and additional exposure history was obtained from the family.

The patient and five family members live together in a rural area of Ayudhaya in central Thailand. Her family reported that she was exposed to several dead chickens in her neighborhood. Neighborhood chickens were noted frequently to roam around the patient’s house, and some had died in front of it. All her family members were well, with no symptoms of influenzalike illness. After the exposure history was obtained, droplet and contact precautions were implemented, and the patient was evaluated for H5N1 influenza. Nasopharnygeal aspirates underwent a rapid influenza A test by enzyme immunomembrane filter assay (Directigen Flu A, Becton Dickinson, Sparks, MD) was negative, and additional tests for viral particles were performed by reverse transcriptase–polymerase chain reaction (RT-PCR), real-time RT-PCR, and viral culture. Because of the negative rapid influenza A test and the lack of access to antiviral medication, neuraminidase inhibitors were not prescribed, but intravenous prednisone was initiated. The patient died from severe adult respiratory distress syndrome (Figure 1B) with multiorgan failure the next day.

**Figure 2:**
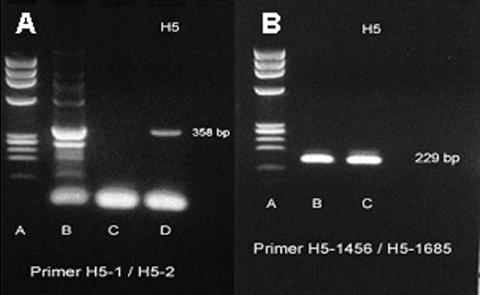
A) reverse transcription–polymerase chain reaction (RT-PCR) specific for H5 gene band (358 bp) of avian influenza H5N1 that was recovered from our patient from nasopharyngeal aspirates by using H5-1/H5-2 primer. Lane A, molecular standard; lane B, H5 band isolated from our patient (358 bp); lane C, negative control; lane D, positive control. B) RT-PCR specific for H5 gene band (229 bp) of avian influenza (H5N1) that was recovered from our patient from nasopharyngeal aspiration by using H5-1456/H5-1685 primer. Lane A, molecular standard; lane B, positive control; lane C, H5 band isolated from our patient (229 bp).

Nasopharnygeal aspirates were positive for influenza A H5 strain by two RT-PCR primers (Figure 2) and by real-time RT-PCR. Three sets of blood cultures, sputum cultures, and serologic tests were negative for the following: *Chlamydia* by microimmunofluorescence, mycoplasma by microparticle agglutination assay, urine *Legionella* antigen by enzyme-linked immunosorbent assay (ELISA), HIV by ELISA, *Burkholderia pseudomallei* (melioid) titer by immunohistochemical assay, dengue titer by hemagglutination inhibition using all four serotypes, *Leptospira* titer by microscopic agglutination test, Widal test, Weil-Felix test, and viral culture. Several dead chickens around the neighborhood near the patient’s house tested positive for H5N1 by viral cultures. The patient’s family declined to permit an autopsy.

Thirty healthcare workers at our hospital and five healthcare workers at the referring hospital were exposed to this index patient without using appropriate personal protective equipment. All of them were monitored for 2 weeks for temporally related influenzalike illness, and temperatures were measured twice weekly. No temporally related influenzalike illness or fever developed. The characteristics and types of exposure reported by influenza A H5N1–exposed healthcare workers are summarized in the Table.

**Table:** Characteristics and types of exposures reported by 35 healthcare workers exposed to avian influenza (H5N1)

Characteristic	No. (%) (N = 35)
Age (median, range; y)	28 (23–34)
Female sex	27 (77)
Type of exposure	
Provided direct patient care	17 (48)
Physical contact	19 (54)
Talked face-to-face	3 (8)
Worked within 1 m	33 (94)
Recalled patient coughing and sneezing	2 (6)
Suctioned respiratory secretions or administered breathing treatment	20 (57)
Changed bed linens	7 (20)
Bathed patient	10 (35)
Temporally related illness^a^	0

^a^Temporally related illness is defined as a respiratory illness that began 1–14 days after exposure to an index patient.

Viral culture for avian influenza H5N1 was conducted on Madin-Darby canine kidney cell monolayers at the Department of Medical Sciences, National Institute of Health, Bangkok. Nasopharyngeal aspiration specimens tested positive by an RT-PCR assay specific for the hemagglutinin gene of influenza A H5N1. The specimen was tested with the primer set for the H5 gene (forward primer H5-1 GCC ATT CCA CAA CAT ACA CCC, reverse primer H5-2 TAA ATT CTC TAT CCT CCT TTC CAA), with an expected product size of 358 bp (8). The specimen was confirmed positive by different RT-PCR primers (forward primer H5-1456 ACG TAT GAC TAT CCA CAA TAC TCA, reverse primer H5-1685 AGA CCA GCT ACC ATG ATT GC), which amplify a DNA fragment of 229 bp. This specimen was further confirmed as positive by using the real-time RT-PCR method, including primer and probe, described by Spackman et al. (11). This assay amplifies a conserved region of European and Asian avian influenza virus and was modified to run on the LightCycler (Roche Molecular Systems, Indianapolis, IN). Precautions for preventing cross-contamination were observed (12).

## Conclusions

The clinical signs and symptoms of avian influenza H5N1 may be more protean than originally described. During the 1997 epidemic in Hong Kong, patients exhibited fever, headache, malaise, myalgia, sore throat, cough, and rhinitis (5,8). Although uncommon, conjunctivitis and gastrointestinal symptoms were also reported (5,8). In the 2004 epidemic in Vietnam, prominent clinical signs and symptoms of avian influenza H5N1 were those of a severe influenza syndrome with fever, cough, diarrhea, and shortness of breath. Of note, diarrhea was present in 7 (70%) of 10 patients along with lower respiratory symptoms (9). The preliminary clinical features of avian influenza H5N1 in the 2004 epidemic in Thailand included fever, cough, sore throat, rhinorrhea, myalgia, and shortness of breath (10). Laboratory findings of patients with severe avian influenza H5N1 are undistinguishable from those of patients with prevailing human influenza; findings include leukopenia, lymphopenia, impaired liver function, prolonged clotting times, and renal impairment (5,8–10). To our knowledge, this patient has the first reported case of H5N1 with fever and gastrointestinal symptoms but no respiratory symptoms.

As of this submission, 22 patients in Vietnam and 12 in Thailand have confirmed cases of avian influenza H5N1. Twenty-three (67%) of 34 infected patients have died. The death rate of H5N1 was 33% (6 of 18 patients) in Hong Kong in 1997, 73% (15 of 22 patients) in Vietnam in 2004, and 67% (8 of 12 patients) in Thailand in 2004. Risk factors associated with severe disease and poor outcome of H5N1 included older age, being symptomatic for a longer period before admission, pneumonia, leukopenia, and lymphopenia (8). Patients <5 years of age had mild disease compared with hospitalized adults (8). However, all cases of H5N1 from Thailand occurred in pediatric patients, except for our patient and one previously reported patient (10). No H5N1 patients in Thailand had underlying or concomitant disease (10).

Avian influenza (H5N1) can be isolated by conventional viral culture methods (1). Several reports suggested that rapid influenza tests, H5-specific RT-PCR, and real-time RT-PCR could aid a rapid diagnosis (1,2,11,13,14). However, rapid diagnostic tests for influenza have low sensitivity, which may limit their usefulness to reliably detect H5N1, especially if illnesses are diagnosed later in their clinical course (1), as in our patient. Thus, clinical findings and a history of poultry exposure may be more helpful in identifying patients with H5N1 infection than the result on rapid diagnostic tests for influenza.

Data are limited on human-to-human transmission of avian influenza H5N1. Whether H5N1 could be efficiently transmitted from human to human is a matter of concern. In a matched case-control study of 15 patients with H5N1, exposure to live poultry in the week before symptom onset was significantly associated with H5N1 disease, while traveling, eating or preparing poultry products, and recent exposure to persons with respiratory illness had no significant association (15). A cohort study conducted among persons infected with H5N1 to detect anti-H5 antibody among their household and social contacts suggests that human-to-human transmission might have occurred through close physical contact with H5N1-infected patients, whereas social exposure to an infected person was not associated with H5N1 infection (16).

Another cohort study, which included healthcare workers from three hospitals where H5N1 patients had been admitted, found a significantly higher rate of seropositivity for H5N1 among exposed workers (8 [3.7%] of 217 persons) than among nonexposed workers (2 [0.7%] of 309 persons), which provides evidence of H5N1 transmission from infected patients to healthcare workers (17). In our study, although 33 (94%) of 35 healthcare workers were exposed to the index patient within 1 m, they did not exhibit fever or influenzalike illness during our 2-week follow-up period (Table). However, we cannot rule out mild or subclinical infection because of the lack of data on anti-H5 serology. Together, these studies confirm that healthcare workers are at low risk of acquiring H5N1 from patients, but continued precautions and monitoring are essential in case the virus evolves to become more transmissible among humans. In conclusion, physicians working in areas where H5N1 is endemic should be aware of unusual cases. A high index of suspicion will facilitate prompt diagnosis and proper management.
